# Silencing of microRNA-517a induces oxidative stress injury in melanoma cells via inactivation of the JNK signaling pathway by upregulating CDKN1C

**DOI:** 10.1186/s12935-019-1064-y

**Published:** 2020-01-29

**Authors:** Chao Yang, Zeqiang Yan, Fen Hu, Wei Wei, Zhihua Sun, Wei Xu

**Affiliations:** 1 Department of Oncology, Xiangyang Central Hospital, Affiliated Hospital of Hubei University of Arts and Science, No. 136, Jingzhou Street, Xiangcheng District, Xiangyang, 441021 Hubei People’s Republic of China; 2 Department of Gastroenterology, Xiangyang Central Hospital, Affiliated Hospital of Hubei University of Arts and Science, Xiangyang, 441021 People’s Republic of China; 3 Department of Dermatology, Xiangyang Central Hospital, Affiliated Hospital of Hubei University of Arts and Science, No. 136, Jingzhou Street, Xiangcheng District, Xiangyang, 441021 Hubei People’s Republic of China

**Keywords:** MicroRNA-517a, Melanoma, CDKN1C, JNK signaling pathway, Oxidative stress

## Abstract

**Background:**

Melanoma is notoriously resistant to current treatments, and less than 25% of metastatic melanoma cases respond to existing therapies. Growing evidence has shown that microRNAs (miRNAs) play a vital role in the prognosis of melanoma. MiR-517a has been implicated in many types of cancer; however, its expressional features and potential biological functions in melanoma remain unclear. The present study aimed to investigate the possible effects of miR-517a on oxidative stress (OS) in melanoma cells.

**Methods:**

miR-517a expression in melanoma was determined using RT-qPCR. After treatment with different concentrations of H_2_O_2_, cell viability was determined in order to identify the most appropriate H_2_O_2_ concentration. Through loss and gain of function experiments, the interactions between miR-517a, the cyclin dependent kinase inhibitor 1C (CDKN1C) and the c-Jun NH2-terminal kinase (JNK) signaling pathway, as well as their roles in OS of melanoma cells were identified. Moreover, the expression of Cleaved Caspase-3, extent of ERK1/2 phosphorylation, Bax/Bcl-2 ratio, levels of T-AOC, ROS and MDA, and SOD activity were also tested. Finally, melanoma cell viability and apoptosis were detected.

**Results:**

MiR-517a was upregulated, while CDKN1C was downregulated in melanoma tissues and cells. MiR-517a targets CDKN1C and consequently reduced its expression. Inhibition of miR-517a was shown to increase Cleaved Caspase-3 expression, Bax/Bcl-2 ratio, levels of ROS and MDA, as well as cell apoptosis but decrease extent of ERK1/2 phosphorylation, T-AOC levels, SOD activity, along with cell proliferation and mitochondrial membrane potential.

**Conclusions:**

Overall, silencing miR-517a results in upregulated CDKN1C expression, and inhibited JNK signaling pathway activation, consequently promoting OS in melanoma cells.

## Background

Melanoma is a type of cancer arising from the melanocyte, which can be found in the skin [1] and digestive tract [2]. It has been previously reported that the key issues for melanoma pathogenesis are genetic factors and ultraviolet radiation exposure [3]. Melanoma is responsible for 80% of skin cancer deaths [4] and its incidence is on the rise in many parts of the world [5]. Accumulated evidence has indicated that oxidative stress (OS) is pathogenic to the progression of melanoma [6] since OS can suppress distant metastasis of melanoma cells in vivo [7]. Eiberger et al. have revealed that OS inhibits the repair of oxidative DNA base modifications in melanoma cells [8]. To date, although therapeutic treatments of melanoma such as chemotherapy, surgery, and immunotherapy were still the common curative options for many patients, however, the 5-year survival rate for post-operation is only 5–10%, due to melanoma metastasis [9, 10]. Therefore, it is important to further investigate new prevention strategies and therapeutic methods for the treatment of melanoma.

In recent years, extensive studies have revealed that microRNAs (miRNAs) play an essential role in the prognosis and metastasis of melanoma [11] in part due to their vital role in a variety of biological processes, such as cellular proliferation, invasion, and apoptosis [12]. Moreover, a recent study showed that miRNAs and their target genes exert a great influence in human melanoma [13]. MiR-517a, on the 19q13.41 locus, is an oncogenic miRNA that promotes cell proliferation and migration, and tumorigenesis [14]. Furthermore, Sun et al. revealed that miR-221 affects the occurrence and progression of colorectal carcinoma (CRC) by targeting cyclin dependent kinase inhibitor 1C (CDKN1C), a key modulator of cell cycle progression [15]. CDKN1C (p57^KIP2^) belongs to the cyclin-dependent kinase (CDK) inhibitors of the Cip/Kip family, which participate in several cellular processes in human cancers [16]. It has been shown that melanoma cells can utilize CDKN1C/P57 to regulate cell cycle arrest [17]. CDKN1C is reported to be a cell-cycle kinase inhibitor and has the potential to inhibit the c-Jun N-terminal kinase (JNK) signaling pathway in chronic inflammatory diseases [18]. JNK is a member of mitogen-activated protein kinases (MAPKs) [19]. Moreover, previous data demonstrated the role of JNK in melanoma, which can offer new targets for melanoma treatment [20]. JNK signaling pathway has emerged as a major regulator of cellular stress responses induced by ultraviolet light, c-irradiation, or pro-inflammatory cytokines, which ultimately lead to cell death [21]. Until now, only a few studies focused on the roles of miR-517a and the JNK signaling pathway via targeting of CDKN1C in OS in melanoma. Therefore, this study was aimed to investigate the possible mechanisms of miR-517a in melanoma through CDKN1C and the JNK signaling pathway.

## Materials and methods

### Ethical statement

All experiments were approved by the Ethical Committee of Xiangyang Central Hospital, Affiliated Hospital of Hubei University of Arts and Science and the written informed consents were obtained from all participants prior to sample collection. All experiments in the present study were conducted in strict accordance with the Declaration of Helsinki.

### Microarray analysis

Based on the melanoma-associated gene expression and miRNA datasets in the Gene Expression Omnibus (GEO) database (https://www.ncbi.nlm.nih.gov/geo/), differentially expressed genes (DEGs) and miRNAs related to melanoma were retrieved, and the molecular mechanism in which they may be involved was speculated. The GSE31909 database contained gene expression data of melanoma cell lines and normal melanocytes. GPL10558-Illumina HumanHT-12 V4.0 expression beadchip was used as the microarray data annotation platform. The GSE35389 dataset contained the expression data in both GPL570-[HG-U133_Plus_2] Affymetrix Human Genome U133 Plus 2.0 Array and GPL8786-[miRNA-1] Affymetrix Multispecies miRNA-1 Array. GPL570 and GPL8786 correspond to the data of gene expression (GSE35389-mRNA) and miRNA expression (GSE35389-miRNA), respectively. The gene expression data of melanoma cells and normal melanocytes were used in differential expression analysis. The Affy package of R language [22] was employed to perform background correction and standardization of pretreatment for microarray data. The limma package of R language [23] was used to screen the DEGs and miRNAs related to melanoma with |log2FC| > 2.0 and adj. p value < 0.01 as the threshold. The jvenn (http://jvenn.toulouse.inra.fr/app/example.html) was used to compare the DEGs of the two gene expression microarray data. The genes related to melanoma were retrieved from DisGeNET (http://www.disgenet.org/web/DisGeNET/menu/search?4) database. The protein interaction analysis was performed using String (https://string-db.org/) database and the Cytoscape 3.6.0 software was used to extract the information of interactions between DEGs and miRNAs related to melanoma, in order to construct the protein–protein interaction (PPI) network. Furthermore, the five prediction tools for miRNA-mRNA relationship, including miRWalk (http://mirwalk.umm.uni-heidelberg.de/), mirDIP (http://ophid.utoronto.ca/mirDIP/), TargetScan (http://www.targetscan.org/vert_71/), miRSearch (http://www.exiqon.com/microrna-target-prediction), and miRpath (http://lgmb.fmrp.usp.br/mirnapath/tools.php) were used to predict the target source miRNAs of DEGs. Finally, jvenn (http://jvenn.toulouse.inra.fr/app/example.html) was performed to compare and analyze the predicted miRNAs.

### Study subjects

We selected 62 patients with melanoma who underwent surgical resection at the Xiangyang Central Hospital, Affiliated Hospital of Hubei University of Arts and Science from January 2015 to February 2018 in this study. There were 32 males and 30 females, aged 21–75 years old, with a mean age of 58.61 ± 9.99 years and a disease course of about 4 ± 1.5 months. The tumor position was observed accordingly: 17 cases of melanoma on the dorsal part of hand, 11 cases of melanoma under the fingernail, 2 cases of melanoma on vulva, 9 cases of melanoma on the head and face, and 23 cases of melanoma on the lower limbs, including 28 cases with lymph node metastasis and 34 cases without lymph node metastasis. According to the Clark classification of skin cancer, 29 cases were stage I, 27 cases were stage II, and 6 cases were stage III. Patients were included in the study if they had complete medical history and clinical data, and no combination of other malignant tumors, treatment with medicine, laser, or radiation therapy. For the normal group, normal skin tissues were collected from a total of 40 patients. This cohort consisted of 23 males and 17 females, aged 15–67 years old, with a mean age of 45.53 ± 10.05 years who received surgical resection during the same period.

### Cell and H_2_O_2_ concentration screening

The human melanoma cell lines A375, G-361, and OCM-1 and, the normal human skin cell line HACAT were used for the following experiments. Among them, G-361 cells were from the American Type Culture Collection (ATCC; Manassas, VA, USA) and the remaining cells were from the Cell Resource Center of the Institute of Basic Medical Sciences, Chinese Academy of Medical Sciences (Beijing, China). Reverse transcription quantitative polymerase chain reaction (RT-qPCR) was employed to screen the melanoma cells with the highest expression of miR-517a. The cells were then cultured in Roswell Park Memorial Institute (RPMI) 1640 culture medium containing 10% serum with 5% CO_2_ at 37 °C, and then sub-cultured.

The cells were treated with H_2_O_2_ to construct a cell model of OS. Cells were treated with 100 μM, 150 μM, 200 μM, 250 μM, and 300 μM H_2_O_2_ that was prepared with serum-free culture medium. After the cell suspension concentration was adjusted to 1 × 10^5^ cells/mL, the cells were inoculated in 96-well plates at a density of 1 × 10^4^ cells per well and incubated overnight. An amount of 100 μL of H_2_O_2_ medium at a final concentration of 250 μmol/L was added into the corresponding wells, with three replicates set in each group. The samples were placed in an incubator for 1 h, 2 h, and 4 h, respectively. After incubation, the absorbance value at 490 nm was measured using a microplate reader in accordance with the instructions of 3-(4,5-dimethylthiazol-2-yl)-2,5-diphenyltetrazolium bromide (MTT) kit. The concentration and time point of H_2_O_2_ with the greatest inhibitory effect on the cells were selected for subsequent experiments.

### Plasmid construction and cell transfection

According to the known sequences of miR-517a and CDKN1C in National Center for Biotechnology Information (NCBI), miR-517a mimic, miR-517a inhibitor, and pcDNA-CDKN1C were constructed by Shanghai Sangon Biotech Co., Ltd., (Shanghai, China). The cells were then transfected with the plasmids of miR-517a inhibitor, pcDNA-CDKN1C, miR-517a mimic + pcDNA-CDKN1C and SP600125 (inhibitor of the JNK signaling pathway).

### Observation of cell morphological changes

After 24 h of transfection, the cells were seeded into 96-well plates at a density of 2 × 10^4^ cells/well, and the morphological changes of the cells were observed under an inverted microscope (IX73, Olympus Corporation, Tokyo, Japan).

### RNA isolation and quantitation

The total RNA from tissues and cells was extracted according to the provided instructions of Trizol reagent (Invitrogen, Carlsbad, CA, USA). The RNA was reverse transcribed into complementary DNA (cDNA) using the PrimeScriptTM RT reagent Kit (Takara, RR047A, Beijing Think-Far Technology Co., Ltd., Beijing, China). The primer sequences (Table 1) were synthetized by Beijing TSINGKE Biological Technology Co., Ltd. (Beijing, China). RT-qPCR was conducted using the ABI 7900HT instrument by the two-step method. With U6 and glyceraldehyde-3-phosphate dehydrogenase (GAPDH) as internal references, the relative gene expression was calculated using the 2^−ΔΔCt^ method. The CT values of miR-517a and U6 expression determined by RT-qPCR were shown in Additional file 1: Tables S1 and S2..

**Table 1:** Primer sequences of related genes for RT-qPCR

Gene	Primer sequence
miR-517a	F: 5′-CGGCGGATCGTGCATCCCTTTA-3′
	R: 5′- GTGCAGGGTCCGAGGT-3′
CDKN1C	F: 5′-AACGCCGAGGACCAGAACC-3′
	R: 5′-GCGAAGAAATCTGCACCGTCT -3′
U6	F: 5′-CTCGCTTCGGCAGCACA-3′
	R: 5′-AACGCTTCACGAATTTGCGT-3′
GAPDH	F: 5′-ATGGAGAAGGCTGGGGCTC-3′
	R: 5′-AAGTTGTCATGGATGACCTTG-3′

*RT-qPCR* reverse transcription quantitative polymerase chain reaction, *miR-517a* microRNA-517a, *CDKN1C* cyclin dependent kinase inhibitor 1C, *GAPDH* glyceraldehyde-3-phosphate dehydrogenase, *F* forward, *R* reverse

### Western blot analysis

Cells were treated with lysis buffer and phosphatase inhibitor (1111111, Beijing Jia Mei Niu Nuo Biotechnology Co., Ltd., Beijing, China) and total protein was collected. Proteins were then separated using 10% sodium dodecyl sulfate–polyacrylamide gel electrophoresis (SDS-PAGE), and transferred onto polyvinylidene fluoride (PVDF) membranes. After blocking with 5% skimmed milk for 1 h, the PVDF membrane was incubated overnight at 4 °C with the diluted primary rabbit antibodies: CDKN1C (1:500, ab75974), JNK (1:2000, ab112501), phosphorylated JNK (phospho T183 + Y185) (1:1000, ab4821), p38 (1:1000, ab27986), phosphorylated p38 (phospho T180 + Y182) (1:1000, ab4822), Cleaved Caspase-3 (1:1000, ab2302), caspase 3 (1:5000, ab32351), ERK1/2 (1:1000, ab17942), phosphorylated ERK1/2 (Thr202/Tyr204) (1:2000, #4370, Cell Signaling Technology, Beverly, MA, USA), Bcl2-associated X protein (Bax) (1:5000, ab32503), and B-cell lymphoma 2 (Bcl-2) (1:2000, ab182858). All abovementioned antibodies were purchased from Abcam Inc. (Cambridge, MA, USA) with the exception of the phosphorylated ERK1/2 antibody. Afterwards, the membrane was washed 3 times with Tris-buffered saline Tween-20 (TBST), incubated with secondary horseradish peroxidase (HRP)-labeled goat anti-rabbit/rat immunoglobulin G (IgG) (HA1003, Shanghai Yanhui Biotechnology Co., Ltd., Shanghai, China) for l h, and immersed in enhanced chemiluminescence (ECL) (ECL808-25, Biomiga, CA, USA). Next, X-ray images were taken (36209ES01, Shanghai Qianchen Biotechnology Co., Ltd., Shanghai, China). The ratio of the gray value of the target band to GAPDH was representative of the relative protein expression.

### Dual-luciferase reporter assay

The wild-type (WT) and mutant (Mut) primers of target predicted CDKN1C 3′ untranslated region (UTR) fragments were designed and synthetized by Shanghai Sangon Biotech Co., Ltd. (Shanghai, China). The pMIR-report luciferase vector was treated with double enzyme digestion using restrictive endonuclease HindIII and PmeI. Next, HindIII/PmeI double enzyme single point was added on both sides of the WT and Mut CDKN1C 3′UTR target predicted fragments. Finally, the target genes were ligated into intended vectors with Ligase 4. CDKN1C 3′UTR-WT-Luc and CDKN1C 3′UTR-Mut-Luc plasmids were co-transfected into 293T cells with the NC mimic and the miR-517a mimic, respectively. Subsequently, the Firefly Luciferase Reporter Gene Assay Kit (RG005, Beyotime Biotechnology Co., Ltd., Shanghai, China) and a microplate reader (MK3, Thermo Fisher Scientific, California, USA) were used to detect luciferase activity at 560 nm.

### 5-ethynyl-2′-deoxyuridine (EdU) staining

The cells were treated with EdU solution, fixed with 40 g/L polyoxymethylene for 30 min, and incubated with glycine solution for 8 min. The cells were then rinsed with PBS containing 0.5% Triton X-100, incubated with Apollo^®^ staining solution, washed with methanol, cultured with Hoechst 3334 solution, and observed under a fluorescence microscope. Three fields of view were selected under 400× magnification. The proliferating cells stained with EdU and total cells stained with Hoechst 33342 were counted. Cell proliferation rate = the number of proliferating cells/total cells × 100%.

### Flow cytometry

Annexin V-fluorescein isothiocyanate (FITC)/propidium iodide (PI) double staining was performed to detect cell apoptosis. The cells were incubated with 5% CO_2_ at 37 °C for 48 h. Subsequently, the cells were suspended in 200 μL binding buffer, added with 10 μL Annexin V-FITC (ab14085, Abcam Inc., Cambridge, MA, USA) and 5 μL PI, and incubated for 15 min. After final resuspension in 300 μL of binding buffer, cell apoptosis was detected on a flow cytometer at an excitation wavelength of 488 nm.

### Mitochondrial membrane potential detection

Mitochondrial membrane potential detection reagent kit (JC-1; C2006) was purchased from Beyotime Biotechnology Co., Ltd. (Shanghai, China). The cells were incubated with 5% CO_2_ at 37 °C for 3 h, added with 500 μL JC-1 working fluid (500 μL 1 × Incubation Buffer + l μL JC-1) in 5% CO_2_ at 37 °C for 15–20 min, rinsed with 1 × Incubation Buffer, and observed under a fluorescence microscope (XSP-63B, Shanghai Optical Instrument Factory, Shanghai, China). The results were scored as follows: normal cells: observation of double color filter: green ++red ++ (high green high red), the color under the same color filter was yellow green; apoptotic cells: observation of double color filter: green ++ red + (high green low red), the color under the same color filter was green.

### Detection of biochemical indexes

The Fe^3+^ reduction method was employed to determine the total antioxidant capacity (T-AOC) using T-AOC reagent kit (YBA015, Shanghai Yu Bo Biological Technology Co., Ltd., Shanghai, China). The reactive oxygen species (ROS) level was determined by fluorescent probe dichloro-dihydro-fluorescein diacetate (DCFH-DA) assay using the ROS reagent kit (50101ES01, Shanghai YEASEN Biological Technology Co., Ltd., Shanghai, China). The xanthine oxidase method was used to detect superoxide dismutase (SOD) activity in accordance to the SOD reagent kit (BC0170, Beijing Solarbio Science & Technology Co., Ltd., Beijing, China). The thiobarbituric acid (TBA) method was used to determine malondialdehyde (MDA) level based on the MDA reagent kit instructions (S0131, Beyotime Biotechnology Co., Ltd., Shanghai, China).

### Statistical analysis

Statistical analysis was performed using the SPSS 21.0 software (IBM Corp. Armonk, NY, USA). Measurement data were expressed as mean ± standard deviation. Comparisons between two groups were analyzed independently by *t*-test, and comparisons among multiple groups were analyzed using one-way analysis of variance (ANOVA). The optical density (OD) value was analyzed using repeated measures ANOVA. The data normality test was conducted using the Kolmogorov–Smirnov method. Comparisons of data with normal distribution among multiple groups were analyzed using ANOVA, followed by Tukey’s post hoc tests with corrections for multiple comparisons. Differences in *p* value of less than 0.05 were considered to be statistically significant.

## Results

### Expression of CDKN1C is altered in melanoma cells

**Fig. 1:**
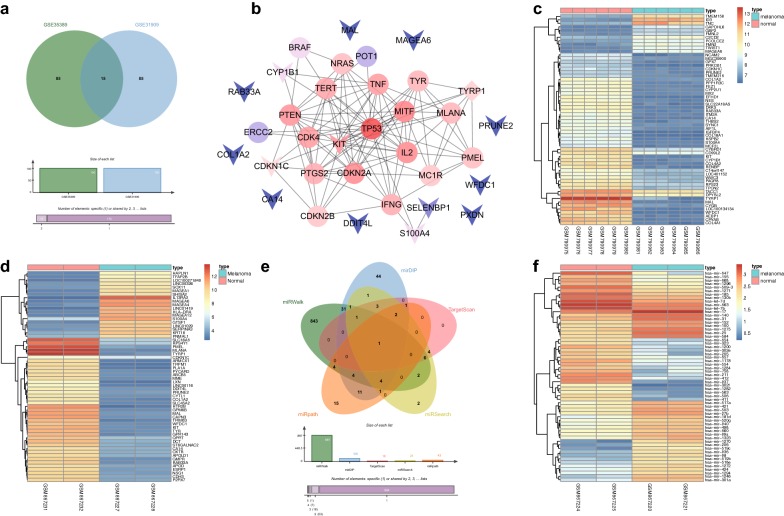
The significance of miR-517a in melanoma. **a** Comparison in the top 100 DEGs from the GSE31909 and GSE35389-mRNA datasets. **b** the PPI network between DEGs and melanoma genes. The arrow represents DEGs, the circle represents the melanoma genes, and the diamond represents the melanoma genes with differential expression. **c**, **d** The heat maps of the top 60 DEGs related to melanoma from the GSE31909 and GSE35389-mRNA microarray data, respectively; the abscissa refers to the sample number, and the ordinate refers to the names of DEGs. The upper right histogram refers to the color gradation. Each block represents the expression level of a gene in one sample. **e** Predicted miRNAs regulating CDKN1C by the miRWalk, mirDIP, TargetScan, miRSearch, and miRpath databases; **f** The heat maps of the top 60 miRNAs with differential expression in GSE35389-miRNA microarray data

Firstly, the R language was used to screen DEGs from melanoma gene expression microarray data (GSE31909 and GSE35389-mRNA). The first 100 DEGs from the GSE31909 and GSE35389-mRNA datasets were selected for comparison and a Venn diagram of the results was constructed (Fig. 1a). The results indicated 15 intersecting genes (MAL, MAGEA6, WFDC1, KIT, TYRP1, S100A4, CDKN1C, COL1A2, DDIT4L, CA14, RAB33A, PRUNE2, PXDN, SELENBP1, and CYP1B1), which were used as melanoma DEGs. The genes related to melanoma were searched for in DisGeNET. The top 20 genes were regarded as melanoma genes (CDKN2A, MC1R, NRAS, MITF, PTEN, TERT, POT1, BRAF, TP53, TYR, CDK4, MLANA, PMEL, IL2, ERCC2, IFNG, TNF, PTGS2, CDKN2B, TYRP1). Based on the protein intersection information in the String database, the PPI network between DEGs and melanoma genes was constructed (Fig. 1b). The DEGs that associated with other genes in this network are KIT, CDKN1C, and TYRP1, suggesting these three DEGs may affect melanoma, in which TYRP1 was both a DEG and a melanoma gene. Moreover, most studies have focused on the effects of KIT on melanoma [24, 25]. Interestingly, the heat maps of the top 60 DEGs of melanoma gene expression microarray data GSE31909 (Fig. 1c) and GSE35389-mRNA (Fig. 1d) revealed that CDKN1C expression in melanoma cells was lower than that in normal melanocytes.

The possible miRNAs regulating CDKN1C were predicted by the miRWalk, mirDIP, TargetScan, miRSearch, and miRpath databases. There were 897 miRNAs with energy < − 20 in miRWalk, and 105 miRNAs were predicted in mirDIP with “Score Class” as “Very High”. In the prediction results of TargetScan, 18 miRNAs were screened with context ++ score < − 0.4 as the threshold, while another 28 and 43 miRNAs were predicted to regulate CDKN1C in miRSearch and miRpath, respectively. After comparing the predicted results of these five miRNAs (Fig. 1e), only one intersecting miRNA (hsa-miR-517a-3p) was found, indicating that miR-517a might regulate CDKN1C. Next, the differentially expressed miRNAs were screened in the melanoma miRNA expression dataset GSE35389-miRNA. The heat maps of the first 60 differential expressed miRNAs were drawn (Fig. 1f), and we noticed that miR-517a expression in melanoma cells was higher than the normal melanocytes. Taken together, these results revealed that miR-517a targets the regulation of CDKN1C-mediated JNK signaling pathway in melanoma.

### Upregulation of miR-517a in melanoma tissues and cells

**Fig. 2:**
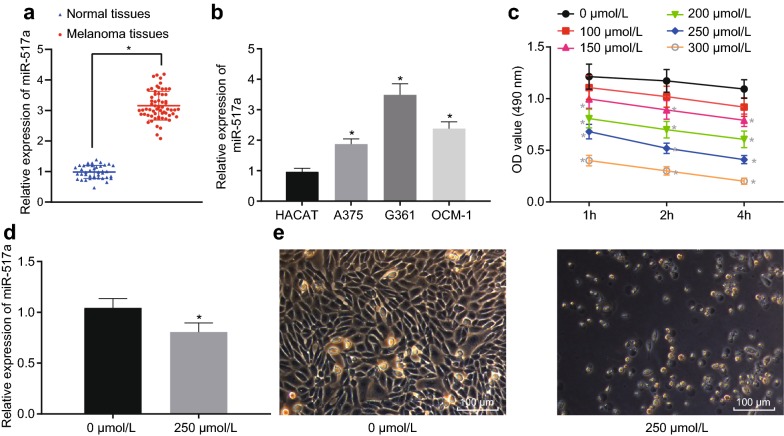
miR-517a is upregulated in melanoma tissues and cells. **a** miR-517a expression in melanoma samples (n = 62) and normal skin tissues (n = 40); *p < 0.05 compared with the normal skin tissues; **b** miR-517a expression in melanoma cells, A375, G-361, and OCM-1 and in normal skin cell, HACAT; **p < 0.05 compared with the HACAT cell; **c** OD value of cells treated with H_2_O_2_ of various concentration for 1 h, 2 h, and 4 h; *p < 0.05 compared with cells treated with 0 μmol/L H_2_O_2_; **d** miR-517a expression after treated with H_2_O_2_; *p < 0.05 compared with cells treated with 0 μmol/L H_2_O_2_; **e** Cell morphology after treatment with 250 μmol/L H_2_O_2_ under the inverted microscope (×400). All data were expressed as mean ± standard deviation; comparisons between two groups were analyzed by unpaired *t*-test; comparisons among multiple groups were analyzed by one-way ANOVA; the OD values at different points were analyzed by repeated measures ANOVA

Next, RT-qPCR was performed to determine miR-517a expression in melanoma tissues and cells. The results showed that miR-517a expression in melanoma samples was significantly increased (*p* < 0.05), as shown in Fig. 2a.

Compared with the HACAT cells, miR-517a was highly expressed in A375, G-361, and OCM-1 cells. Among them, G-361 cells showed the highest miR-517a expression (*p* < 0.05), as shown in Fig. 2b. Thus, G-361 cells were selected for subsequent experiments.

The cells were treated with 100 μM, 150 μM, 200 μM, 250 μM, 300 μM of H_2_O_2_ to construct OS cell models. The results of H_2_O_2_ concentration screening (Fig. 2c) indicated the survival rate of cells gradually decreased with the increase of H_2_O_2_ concentration and H_2_O_2_ treatment time. When H_2_O_2_ concentration was about 250 μmol/L and the processing time was 2 h, the proliferation rate of cells was about 50%, which was the lowest concentration and shortest time to minimize the half growth of cells. Therefore, 250 μmol/L H_2_O_2_ was used to treat the subsequent cells for 2 h.

The expression of miR-517a was detected by RT-qPCR, and the results showed that, when compared with the untreated cells, the miR-517a expression was significantly decreased in cells treated with 250 μmol/L H_2_O_2_ (*p* < 0.05) (Fig. 2d).

The results observed under the microscope (Fig. 2e) showed that when compared with the untreated cells, treatment with 250 μmol/L H_2_O_2_ resulted in more obvious cytoplasmic shrinkage, sparse cells, and mainly round cells. These results revealed that miR-517a was upregulated in melanoma tissues and cells, while the expression of miR-517a was significantly decreased after treatment with H_2_O_2_ (250 μmol/L).

### miR-517a regulates OS damage in melanoma cells

**Fig. 3:**
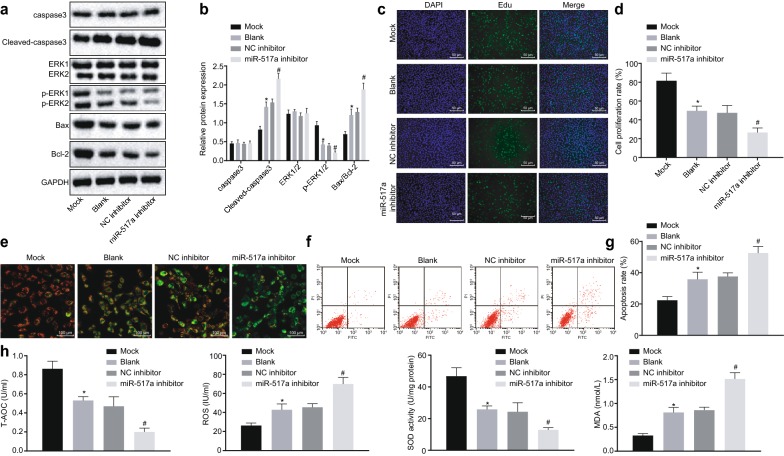
Silencing miR-517a leads to OS in melanoma cells. **a**, **b** Western blot analysis of Cleaved Caspase-3, phosphorylated-ERK2 and Bax/Bcl-2 in G-361 cells treated with 250 μmol/L H_2_O_2_; **c** EdU staining of G-361 cells treated with 250 μmol/L H_2_O_2_ (×200). **d** Proliferation rate of cells treated with 250 μmol/L H_2_O_2_. **e** Cell membrane potential changes of cells treated with 250 μmol/L H_2_O_2_ under the fluorescence microscope (×400). **f**, **g** apoptosis rate of cells treated with 250 μmol/L H_2_O_2_. **h** Levels of T-AOC, ROS, and MDA along with SOD activity in cells treated with 250 μmol/L H_2_O_2_. *p < 0.05 compared with cells without treatment; ^#^*p* < 0.05 compared with cells treated with NC inhibitor. All data were expressed as mean ± standard deviation; comparisons among multiple groups were analyzed by one-way ANOVA; the experiment was repeated three times

G-361 cells were treated with or without H_2_O_2_, or transfected with miR-517a inhibitor sequence, pcDNA-CDKN1C sequence, miR-517a mimic + pcDNA-CDKN1C sequence, SP600125 (JNK inhibitor), or relative negative control (NC) individually or together to identify their roles in OS of melanoma cells. Western blot analysis (Fig. 3a, b), EdU (Fig. 3c, d), and flow cytometry (Fig. 3f, g) revealed that in the cells treated with 250 μmol/L H_2_O_2_, the protein levels of Cleaved Caspase-3, ratio of Bax/Bcl-2 as well as cell apoptosis rate were significantly enhanced while extent of ERK1/2 phosphorylation and cell proliferation were decreased when compared to the cells without treatment (all *p* < 0.05). Compared with the cells treated with NC inhibitor, the protein levels of Cleaved Caspase-3, ratio of Bax/Bcl-2 and cell apoptosis rate were significantly increased, while the extent of ERK1/2 phosphorylation and cell proliferation were reduced in the cells treated with miR-517a inhibitor and H_2_O_2_ (*p* < 0.05).

As shown in Fig. 3e, after treatment with 250 μmol/L of H_2_O_2_, the normal cells showed red and green fluorescence, presented with yellow-green color, and showed no significant changes in the membrane potential. In contrast, apoptotic cells presented with high green and low red in color, and the membrane potential was significantly decreased. The normal cells showed no changes in the membrane potential, and the results in cells treated with H_2_O_2_ and NC inhibitor showed no significant difference. In contrast to cells treated with NC inhibitor, the membrane potential in cells treated with H_2_O_2_ and miR-517a inhibitor was significantly decreased (*p* < 0.05).

The levels of biochemical indexes in G-361 cells, including T-AOC, ROS, and MDA as well as SOD activity, were measured. As shown in Fig. 3h, cells treated with H_2_O_2_ (250 μM) showed elevated ROS and MDA levels, and reduced T-AOC levels and SOD activity in comparison to the cells without treatment (all *p* < 0.05). When compared with cells treated with NC inhibitor, the cells treated with miR-517a inhibitor exhibited elevated ROS and MDA levels and reduced T-AOC levels and SOD activity (all *p* < 0.05). The abovementioned findings revealed that silencing miR-517a promoted OS of G-361 cells.

### miR-517a inhibition suppresses activation of the JNK signaling pathway by elevating CDKN1C

**Fig. 4:**
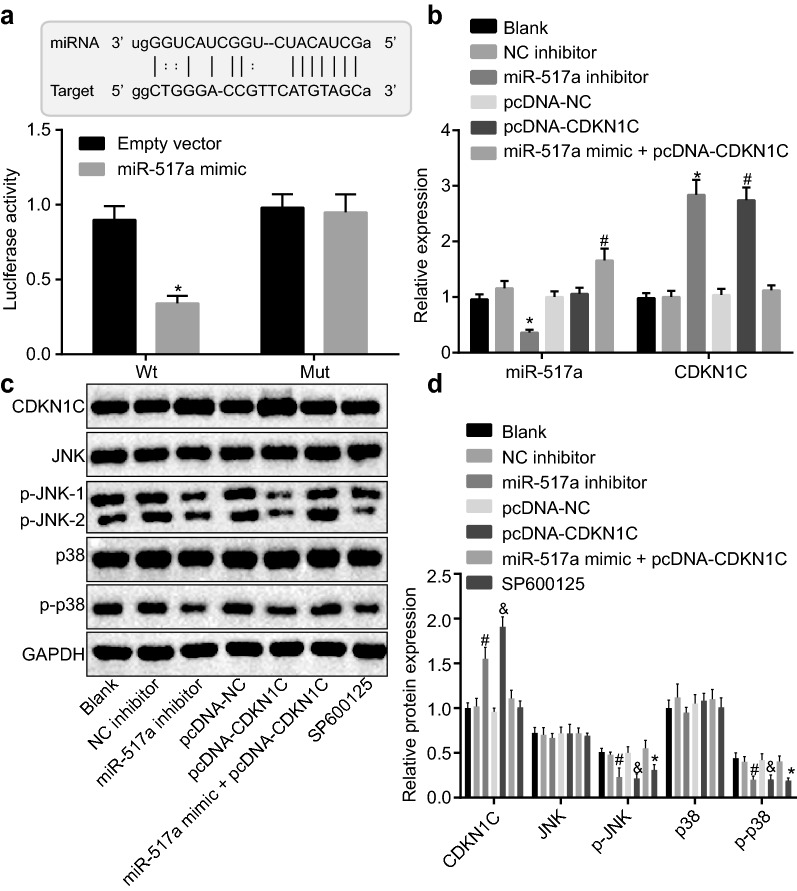
miR-517a inhibition suppressed activation of the JNK signaling pathway by targeting CDKN1C. **a** Binding of miR-517a to CDKN1C confirmed by dual-luciferase reporter assay; *p < 0.05 compared with cells treated with empty vector. **b** miR-517a expression and mRNA level of CDKN1C in cells detected using RT-qPCR. **c**, **d** Western blot analysis of CDKN1C, JNK, and p38 proteins in cells. *p < 0.05 compared with cells without treatment; ^#^*p* < 0.05 compared with cells treated with NC inhibitor; ^&^*p* < 0.05, compared with the cells treated with pcDNA-NC. All data were expressed as mean ± standard deviation; comparisons among multiple groups were analyzed by one-way ANOVA; the experiment was repeated three times

Dual-luciferase reporter assay was used to identify the target relationship between miR-517a and CDKN1C. After co-transfection with CDK N1C-WT-Luc plasmid into 293T cells, the luciferase activity was significantly lower in cells transfected with miR-517a mimic than that of the empty vector-transfected cells (*p *< 0.05) (Fig. 4a).

RT-qPCR was used to determine miR-517a expression and CDKN1C mRNA expression. As shown in Fig. 4b, the cells treated with miR-517a mimic + pcDNA-CDKN1C displayed increased miR-517a expression (*p *< 0.05) when compared to cells without treatment, while cells treated with NC inhibitor, pcDNA-NC, and miR-517a mimic + pcDNA-CDKN1C showed no significant changes in the CDKN1C mRNA levels (all *p* > 0.05). The cells treated with miR-517a inhibitor presented with a downregulated miR-517a expression, and upregulated CDKN1C mRNA levels (both *p *< 0.05) when compared with cells treated with NC inhibitor. The cells treated with pcDNA-CDKN1C showed no significant difference in miR-517a expression, but CDKN1C mRNA level was increased when compared with the cells treated with pcDNA-NC (*p *< 0.05).

Several studies have indicated that activation of the JNK signaling pathway is involved in melanoma [26, 27], and CDKN1C is known to inhibit the JNK signaling pathway [28], suggesting that CDKN1C could modulate the JNK signaling pathway in melanoma. Western blot analysis showed that the levels of JNK and p38 were not affected by miR-517a and CDKN1C. Compared with cells without treatment, the extent of JNK and p38 phosphorylation was decreased in the cells treated with SP600125, while the cells treated with NC inhibitor, pcDNA-NC, and miR-517a mimic + pcDNA-CDKN1C showed no significant difference (all *p* > 0.05). Compared with cells treated with NC inhibitor, CDKN1C protein was upregulated and the extent of JNK and p38 phosphorylation was decreased in the cells treated with miR-517a inhibitor (all *p *< 0.05). Compared with cells treated with pcDNA-NC, the cells treated with pcDNA-CDKN1C showed upregulated CDKN1C protein expression and decreased extent of JNK and p38 phosphorylation (all *p *< 0.05) (Fig. 4c, d).

The abovementioned findings revealed a target relationship between miR-517a and CDKN1C, the downregulation of which inhibited activation of the JNK signaling pathway.

### CDKN1C/JNK signaling pathway regulates melanoma cells to exert anti-oxidative stress

**Fig. 5:**
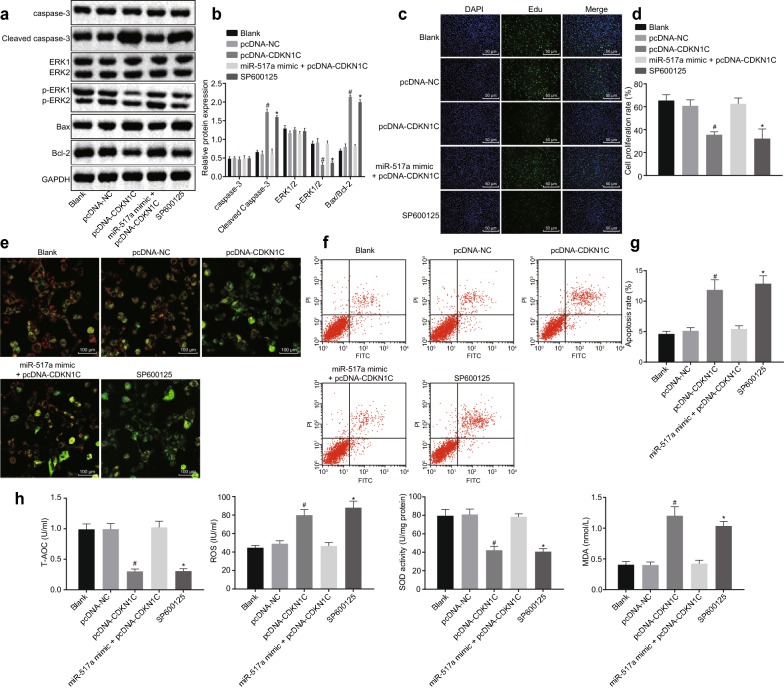
MiR-517a inhibition enhances OS of cells via upregulation of CDKN1C and suppression of the JNK signaling pathway. **a**, **b** Western blot analysis of Cleaved Caspase-3, Bax/Bcl-2 ratio and extent of ERK2 phosphorylation in cells treated with 250 μmol/L H_2_O_2_. **c** EdU staining of cells treated with 250 μmol/L H_2_O_2_ (×200). **d** Proliferation rate of cells treated with 250 μmol/L H_2_O_2_. **e** Membrane potential changes of cells treated with 250 μmol/L H_2_O_2_ under a fluorescence microscope (×400). **f**, **g** Apoptosis rate of cells treated with 250 μmol/L H_2_O_2_. **h** Levels of T-AOC, ROS, and MDA along with SOD activity in cells treated with 250 μmol/L H_2_O_2_; *p < 0.05 compared with cells without treatment; ^#^*p* < 0.05 compared with cells treated with NC inhibitor. All data were expressed as mean ± standard deviation; comparisons among multiple groups were analyzed by one-way ANOVA; the experiment was repeated three times

Western blot analysis was performed to determine the protein levels of Cleaved Caspase-3, Bax/Bcl-2 ratio and the extent of ERK1/2 phosphorylation in cells following different treatments. As shown in Fig. 5a, b, compared to untreated cells, the protein levels of Cleaved Caspase-3 and Bax/Bcl-2 ratio were significantly elevated while the extent of ERK1/2 phosphorylation was reduced in the cells treated with SP600125 (all *p* < 0.05). Compared with cells treated with pcDNA-NC, Cleaved Caspase-3 protein levels and Bax/Bcl-2 ratio were significantly increased while the extent of ERK1/2 phosphorylation was decreased in cells treated with pcDNA-CDKN1C (all *p* < 0.05).

EdU staining was performed to detect cell proliferation after cells were treated with 250 μmol/L H_2_O_2_. As shown in Fig. 5c, d, compared with cells without treatment, cell proliferation was significantly inhibited in cells treated with SP600125 (*p* < 0.05). The cells treated with pcDNA-CDKN1C showed an inhibited cell proliferation when compared to the cells treated with pcDNA-NC (*p* < 0.05).

Mitochondrial membrane potential detection was also conducted after treatment with 250 μmol/L H_2_O_2_. As shown in Fig. 5e, the cells with normal growth showed red and green fluorescence, the cells presented yellow green, and showed no significant changes in membrane potential. In contrast, apoptotic cells showed high green and low red in color, and the membrane potential was significantly decreased. Cells without treatment, as well as cells treated with pcDNA-NC, and miR-517a mimic + pcDNA-CDKN1C displayed no significant changes in membrane potential. Compared to untreated cells, the membrane potential was decreased in cells treated with SP600125 (*p* < 0.05). Compared to cells treated with pcDNA-NC, the membrane potential was significantly reduced in cells treated with pcDNA-CDKN1C (*p* < 0.05).

Flow cytometry was performed to detect cell apoptosis upon treatment with 250 μmol/L H_2_O_2_. As shown in Fig. 5f, g, compared with cells without treatment, cell apoptosis was increased in cells treated with SP600125 (*p* < 0.05). Cells treated with pcDNA-CDKN1C showed enhanced apoptosis compared to cells treated with pcDNA-NC (*p* < 0.05).

The levels of biochemical indexes in G-361 cells, including T-AOC, ROS, and MDA as well as SOD activity, were measured after treatment with 250 μmol/L H_2_O_2_. As shown in Fig. 5h, the cells treated with SP600125 showed elevated ROS and MDA levels but decreased T-AOC levels and SOD activity compared to cells without treatment (all *p* < 0.05). Compared with cells treated with pcDNA-NC, the cells treated with pcDNA-CDKN1C exhibited elevated ROS and MDA levels and reduced T-AOC levels and SOD activity (all *p* < 0.05). The abovementioned findings revealed that overexpression of CDKN1C promoted OS in melanoma G-361 cells. At the same time, upregulation of miR-517a could reverse the effects of CDKN1C, and the suppression of the JNK signaling pathway could promote OS in melanoma cells.

## Discussion

Melanoma is a highly aggressive and prevalent skin cancer with high mortality, and OS and DNA damage due to ultraviolet light have been identified as the leading causes of melanoma formation [29]. In recent years, the incidence of melanoma has been increasing globally [30]. Numerous studies have revealed that miRNAs are expressed abnormally in a variety of human diseases, including melanoma [13, 31]. Our work aimed to assess the mechanism of miR-517a in melanoma. It was demonstrated that the inhibition of miR-517a contributes to OS via inactivation of the JNK signaling pathway by upregulating CDKN1C, in melanoma cells.

Initially, our data demonstrated that miR-517a was upregulated, while CDKN1C was downregulated in melanoma cells. A previous study identified that about half of miRNAs are located in chromosomal areas, known to amplify or delete in human cancer cells [32]. miR-517a is also known as an oncomiR in hepatocellular carcinoma (HCC) with elevated expression in HCC samples and, it can increase proliferation, migration, and invasion of HCC cells in vitro as well as drive tumorigenesis and metastasis in vivo [14]. Furthermore, high hsa-miR-517a expression is a contributor to the worse prognosis for tumor progression/recurrence-free survival and, overall survival of patients with glioblastoma [33]. Overexpression of miR-517a-3p in lung cancer cell lines has been found to increase lung cancer cell proliferation, migration and invasion abilities through inhibition of forkhead box J3 (FOXJ3) expression [34]. Furthermore, miR-517a is detected to be activated in CRC, and is related to poor prognosis and low survival rates of CRC patients [35]. It has been reported that miRNAs have the ability to promote tumor proliferation and metastasis by downregulating the expression of their target genes [36]. Our study showed that CDKN1C was a target gene of miR-517a and its expression can be downregulated by miR-517a. It has been shown that miR-221 is involved in CRC by targeting CDKN1C [15]. A recent study has also shown that CDKN1C exhibits reduced mRNA expression in several human cancers [37]. CDKN1C, known as a tumor suppressor gene, is found to be decreased in patients with cutaneous T-cell lymphoma, which is the most common lymphoma of the skin [38]. CDKN1C suppression following CITED1 overexpression has been shown to augment cell cycle progression and cell viability in melanoma cells [17].

In addition, this study also confirmed that inhibition of miR-517a resulted in increased levels of CDKN1C, Cleaved Caspase-3 and Bax/Bcl-2 ratio, as well as reduced the extent of ERK1/2, JNK and p38 phosphorylation, suggesting that silencing of miR-517a inhibited cell proliferation and promoted apoptosis via upregulation of CDKN1C and inactivation of the JNK signaling pathway. Bax has been reported to induce apoptosis [39], while Bcl-2 serves as an apoptosis inhibitor [40]. A previous study reported that suppression of miR-150 promotes apoptosis of melanoma cells [41]. Moreover, miR-221 was shown to promote colon carcinoma cell proliferation by downregulating CDKN1C/p57 expression [42]. In addition, miRNA could also affect JNK expression [43]. Beales et al. have shown that activation of JNK inhibits cell apoptosis [44], suggesting that the suppression of JNK enhances cell apoptosis. A previous study has also demonstrated that inhibition of JNK could facilitate cell apoptosis [26]. However, the role of the miR-517a/CDKN1C/JNK signaling in melanoma still remains enigmatic. Thus, further investigations are needed to further explore their effects on melanoma.

Interestingly, this study has found that the miR-517a silencing or SP600125 resulted in increased levels of ROS and MDA, and decreased levels of T-AOC and SOD activity. OS often occurs in the progression of melanoma [6]. OS contributes to increased levels of ROS including H_2_O_2_ [45, 46]. ROS usually generates from the process of metabolic processes in healthy cells, but is also produced in malignant melanoma [47]. MDA is also considered to be a marker of OS that can be determined in the blood [48]. In addition, elevated OS levels are reported to be correlated with reduced levels of T-AOC [49]. SOD is one of the primary antioxidant enzymes that exist in mammalian cells [50]. Wang et al. revealed that upregulation of SOD1 could suppress OS [51]. MiR-141 was identified to attenuate OS in human retinal ganglion cells [52]. Furthermore, activity of the JNK signaling pathway is capable of alleviating the toxic effects of ROS [53]. These findings suggest that the downregulation of miR-517a promotes OS in melanoma cells by suppressing activation of the JNK signaling pathway and targeting CDKN1C.

## Conclusions

**Fig. 6:**
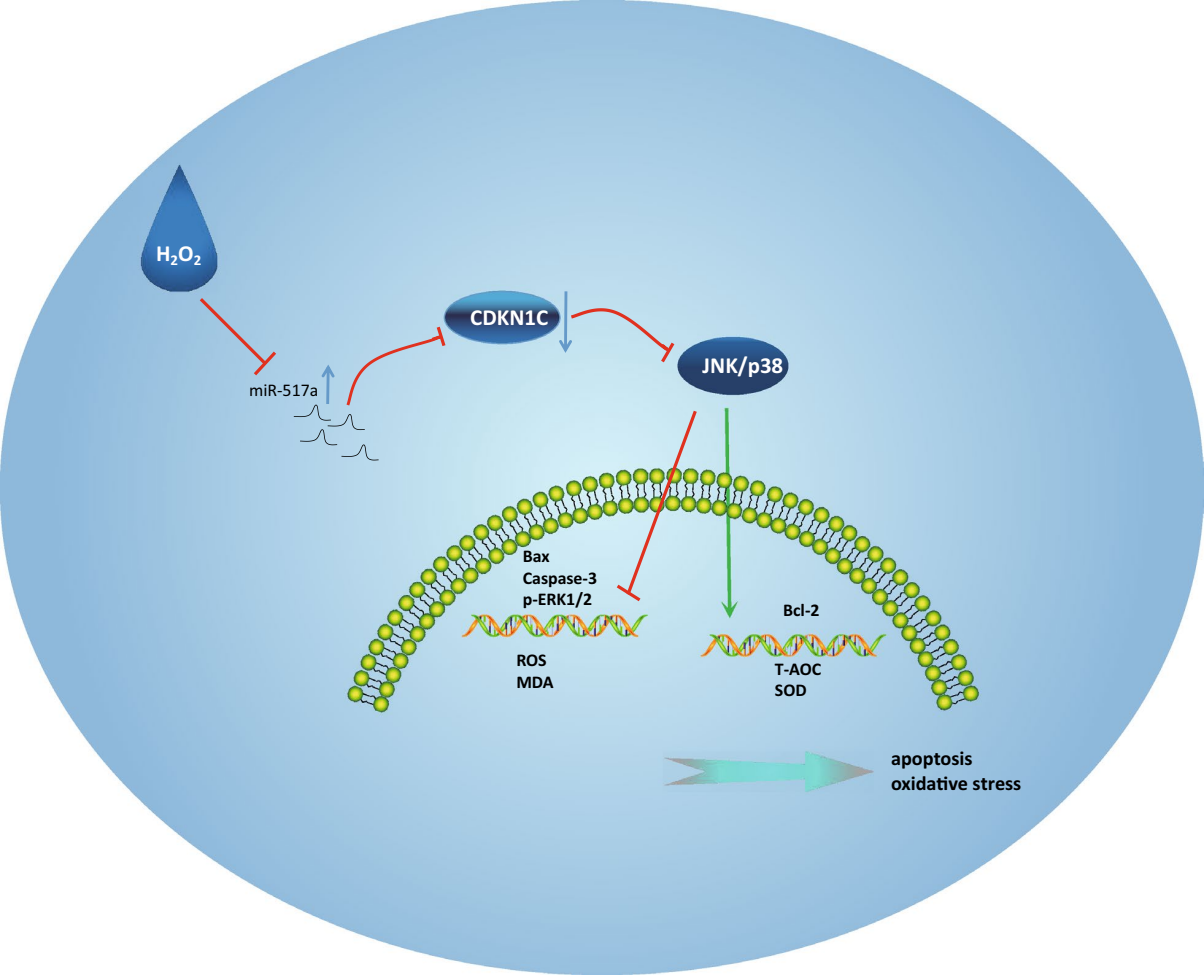
The mechanisms of the regulatory network and function of miR-517a in OS in melanoma. miR-517a was highly expressed, while CDKN1C was downregulated. H_2_O_2_ could inhibit the miR-517a expression and the JNK/p38 signaling pathway to affect the levels of apoptosis-related factors and OS-related factors, ROS and MDA

In conclusion, we show here that miR-517a is activated in melanoma and that it directly targets CDKN1C. Downregulation of miR-517a inactivated the JNK signaling pathway while upregulating CDKN1C expression, leading to OS in melanoma (Fig. 6). Our work thus identifies the potential role of miR-517a/CDKN1C/JNK axis in the treatment of OS in melanoma. Further investigations into the interaction between miR-517a, CDKN1C and the JNK signaling pathway are still required to fully understand the specific mechanisms of miR-517a in melanoma.

## Supplementary information

**Additional file 1: Table S1.** CT values of miR-517a and U6 expression determined by RT-qPCR in Fig. 2b. **Table S2.** CT values of miR-517a and U6 expression determined by RT-qPCR in Fig. 2d.

## Data Availability

All data generated or analyzed during this study are included in this article.

## Abbreviations

miRNAs: microRNAs
OS: oxidative stress
CDKN1C: cyclin dependent kinase inhibitor 1C
JNK: c-Jun NH2-terminal kinase
CRC: colorectal carcinoma
CDK: cyclin-dependent kinase
MAPKs: mitogen-activated protein kinases
GEO: Gene Expression Omnibus
DEGs: differentially expressed genes
PPI: protein-protein interaction
ATCC: American Type Culture Collection
RPMI: Roswell Park Memorial Institute
RT-qPCR: reverse transcription quantitative polymerase chain reaction
SDS-PAGE: sodium dodecyl sulfate-polyacrylamide gel electrophoresis
MTT: 3-(4,5-dimethylthiazol-2-yl)-2,5-diphenyltetrazolium bromide
NCBI: National Center for Biotechnology Information
cDNA: complementary DNA
GAPDH: glyceraldehyde-3-phosphate dehydrogenase
PVDF: polyvinylidene fluoride
TBST: tris-buffered saline Tween-20
HRP: horseradish peroxidase
Bax: Bcl2-associated X protein
Bcl-2: B cell lymphoma 2
HRP: horseradish peroxidase
IgG: immunoglobulin G
ECL: enhanced chemiluminescence
WT: wild-type
Mut: mutant
UTR: untranslated region
FITC: fluorescein isothiocyanate
PI: propidium iodide
T-AOC: total antioxidant capacity
ROS: reactive oxygen species
SOD: superoxide dismutase
MDA: malondialdehyde
TBA: thiobarbituric acid
EdU: 5-ethynyl-2′-deoxyuridine
ANOVA: analysis of variance
OD: optical density
NC: negative control
