# Supplementary material for: Silencing of microRNA-517a induces oxidative stress injury in melanoma cells via inactivation of the JNK signaling pathway by upregulating CDKN1C
Source: Cancer Cell Int. 2020 Jan 29;20:32. doi: 10.1186/s12935-019-1064-y (PMC6990552; doi:10.1186/s12935-019-1064-y)
Supplement: Supplementary file 1 — Additional file 1: Table S1. CT values of miR-517a and U6 expression determined by RT-qPCR in Fig. 2b. Table S2. CT values of miR-517a and U6 expression determined by RT-qPCR in Fig. 2d. [file 12935_2019_1064_MOESM1_ESM.docx]

**Additional file 1: Table S1.** CT values of miR-517a and U6 expression determined by RT-qPCR in Figure 2B

| Sample | Ct (miR-517a ) | Ct (U6) |
| --- | --- | --- |
| HACAT | 18.647952130 | 17.742982864 |
| HACAT | 18.942728240 | 18.564216614 |
| HACAT | 17.252982860 | 17.162982864 |
| A375 | 20.427258560 | 18.520847321 |
| A375 | 20.058874791 | 18.931901932 |
| A375 | 18.348401982 | 17.306068420 |
| G361 | 21.129147573 | 18.570659637 |
| G361 | 20.231181260 | 17.902574539 |
| G361 | 19.427584091 | 17.546812057 |
| OCM-1 | 20.210444039 | 18.082670212 |
| OCM-1 | 20.227638037 | 18.717536926 |
| OCM-1 | 19.002065124 | 17.520847321 |

**Additional file 1: Table S2.** CT values of miR-517a and U6 expression determined by RT-qPCR in Figure 2D

| Sample | Ct (miR-517a ) | Ct (U6) |
| --- | --- | --- |
| 0 μmol/L | 19.147952130 | 18.823391564 |
| 0 μmol/L | 18.337940615 | 17.106403988 |
| 0 μmol/L | 19.410619734 | 18.431274692 |
| 250 μmol/L | 17.248145409 | 17.216943786 |
| 250 μmol/L | 19.027487151 | 17.962453187 |
| 250 μmol/L | 17.647774450 | 17.160507943 |
